# Role of endoplasmic reticulum autophagy in acute lung injury

**DOI:** 10.3389/fimmu.2023.1152336

**Published:** 2023-05-17

**Authors:** Shiping Liu, Xiaoyu Fang, Ruiyao Zhu, Jing Zhang, Huijuan Wang, Jiaxi Lei, Chaoqun Wang, Lu Wang, Liying Zhan

**Affiliations:** ^1^ Department of Critical Care Medicine, Renmin Hospital of Wuhan University, Wuhan, China; ^2^ Department of Infection Prevention and Control, Renmin Hospital of Wuhan University, Wuhan, China; ^3^ College of Life Sciences, Wuhan University, Wuhan, China

**Keywords:** ER-phagy, ER-phagy receptor, ALI, ARDS, immunity, inflammation, ER stress

## Abstract

Acute lung injury (ALI) and acute respiratory distress syndrome (ARDS), the prime causes of morbidity and mortality in critically ill patients, are usually treated by general supportive treatments. Endoplasmic reticulum autophagy (ER-phagy) maintains cellular homeostasis by degrading damaged endoplasmic reticulum (ER) fragments and misfolded proteins. ER-phagy is crucial for maintaining ER homeostasis and improving the internal environment. ER-phagy has a particular role in some aspects, such as immunity, inflammation, cell death, pathogen infection, and collagen quality. In this review, we summarized the definition, epidemiology, and pathophysiology of ALI/ARDS and described the regulatory mechanisms and functions of ER-phagy as well as discussed the potential role of ER-phagy in ALI/ARDS from the perspectives of immunity, inflammation, apoptosis, pathogen infection, and fibrosis to provide a novel and effective target for improving the prognosis of ALI/ARDS.

## 1 Introduction

Acute lung injury (ALI) and acute respiratory distress syndrome (ARDS) are associated with various lung injuries that often lead to life-threatening consequences. Developed from ALI, ARDS was first formally described in 1967 (1). Recently, ALI has been defined as mild or moderate ARDS (2). ARDS is an acute respiratory failure characterized by severe hypoxemia caused by noncardiogenic pulmonary edema. There are multiple etiologies of ARDS, classified as pulmonary endogenous and exogenous, and the most common causes are pneumonia and sepsis (3). The pathophysiology of ARDS is divided into exudative, proliferative, and fibrotic stages. During the exudative phase, reduced lung compliance leads to gas exchange impairment. The proliferation phase is the recovery period of the lung microvascular barrier, while the fibrosis phase leads to poor recovery of lung injury and increased ARDS mortality (4). Due to the poor effects of prevention and treatment, ALI/ARDS has exceptionally high morbidity and mortality. In 2016, a study on the incidence of ARDS in intensive care units (ICUs) in 50 countries showed that 10% of ICU patients and 23% of mechanically ventilated patients met the diagnostic criteria for ARDS (5). The mortality in hospitals for ARDS is 35%-45%, and a significant proportion of ARDS mortality is attributed to sepsis-associated ARDS (6). ALI progression is associated with various clinical disorders, leading to diverse outcomes. Sepsis is the main cause of ALI death, and the mortality rate is up to 43% (2). Moreover, the prognosis of ALI/ARDS is unsatisfactory, and most patients will face sequelae, such as muscle weakness, physical deterioration, and even cognitive impairment (7).

The ER, the largest organelle in the cell, is responsible for lipid and protein biosynthesis, and provides important sites for modifying and folding nascent integral membranes and secreted proteins. However, under some pathological stimuli, ER function is disrupted, leading to the accumulation of misfolded proteins, which triggers ER stress. During ER stress, cells activate a series of complementary mechanisms in response to changes in protein folding, a process known as the unfolded protein response (UPR) (8). UPR is a highly conserved mechanism mediated by three proteins sensors located in ER: protein kinase RNA (PKR)-like ER kinase (PERK), inositol-requiring enzyme-1 (IRE-1), and activating transcription factor-6 (ATF6). ER stress induces ER-phagy by activating the UPR pathway (9). This phenomenon was first discovered in a yeast experiment in 2007, in which researchers found that ER stress induces ER selective autophagy, degrading dysfunctional ER membranes and restoring ER homeostasis (10). ER-phagy contains two primary autophagy mechanisms, namely, macroautophagy and microautophagy. The mechanism of macroautophagy is that the autophagy bilayer membrane extends and wraps the endoplasmic reticulum fragments, finally binding to the lysosomes and being degraded. Microautophagy is the process by which the lysosomal membrane undergoes invaginations and extrudes part of the endoplasmic reticulum into the lysosome (11).

Restricted treatments lead to poor prognosis of ALI/ARDS, and clarification of the pathogenesis is critical for treatment. Therefore, an in-depth understanding of the pathogenesis of ALI/ARDS is essential. In this review, we discussed the possible role of ER-phagy in ALI/ARDS, which may provide new insights for treating ALI/ARDS, thus reducing ALI/ARDS incidence and improving ALI/ARDS prognosis.

## 2 Regulatory mechanism of ER-phagy

Several studies have suggested that ER-phagy receptors are the primary regulatory mechanism of ER-phagy. To induce ER-phagy, late-stage ER stress activates ER-phagy receptors, which interact with the light chain (LC3)/GABARAP (GABA type A receptor-associated protein)/autophagy protein (ATG)8 autopahgy protein complex. In addition, recent studies have also found that complexes of mitochondrial oxidative phosphorylation (OXPHOS) and DDRGK1-mediated ER surface UFMylation are closely associated with ER-phagy. Therefore, mitochondrial factors and DDRGK1 proteins are also involved in regulating ER-phagy.

### 2.1 ER-phagy receptors

**Figure 1:**
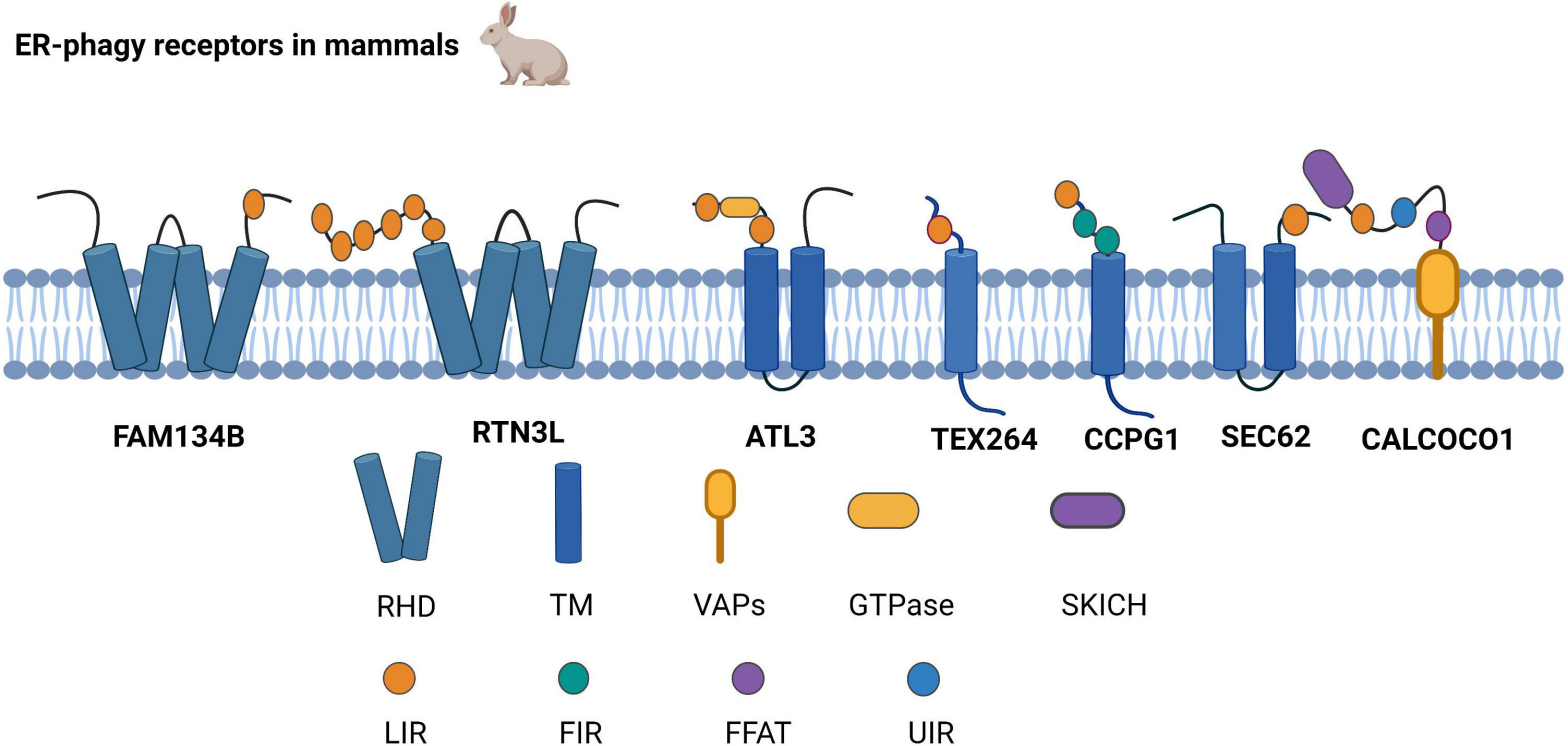
Structure of ER-phagy Receptors in Mammals. ER-phagy receptors, distributed in a specific ER domain, bind to autophagic proteins and then assemble into autophagosomes, thus recruiting the autophagy machinery. Mammalian ER-phagy receptors are mainly FAM134B, RTN3L, ATL3, TEX264, CCPG1, and SEC62, and the novel receptor CALCOCO1 has been found to mediate ER-phagy currently.

ER-phagy receptors are an essential part of ER-phagy, including the family with sequence similarity 134 member B (FAM134B), reticulon-3L (RTN3L), atlastin GTPase 3 (ATL3), testis expressed 264 (TEX264), cell cycle progression 1 (CCPG1), and preprotein translocation factor SEC62, coiled-coil domain-containing protein 1 (CALCOCO1),which bind to autophagy-related proteins and mediate ER-phagy (**Figure 1**).

#### 2.1.1 FAM134B

FAM134B is currently the most widely studied autophagy receptor, and it has two significant structures, LIR and RHD. LIR binds to the LC3/GABARAP family of autophagy proteins and is a typical structure of selective autophagy receptors (12, 13). RHD has two hairpin structures, namely, TM1-TM2 and TM3-TM4, which are responsible for anchoring the protein to the ER membrane, subsequently recognizing and inducing the bending of the ER membrane (11). Cinque et al. found that promoting TFEB/transcription factor 3 (TFE3) nuclear translocation by activating fibroblast growth factor (FGF) signaling facilitates the transcription of FAM134B (14). Because ATG5 and Beclin1 are valuable in FAM134B-mediated ER-phagy, it has been demonstrated that FAM134B depends on macro-ER-phagy (11).

#### 2.1.2 RTN3L

RTN3L is generally localized to the endoplasmic reticulum through the RHD structure. RTN3 interacts with LC3/GABARAP *via* the LIR. As an ER-phagy receptor, RTN3L in the oligomeric state promotes ER tubule fragment formation, and the functional LIR motif of RTN3L then promotes the transport of ER debris into lysosomes (15). Misfolded proteins escape the ER by damaging the ER membrane, while RTN3L protects ER membrane integrity based on ER-phagy (16). Furthermore, RTN3L maintains homeostasis in immunity and inflammation by disrupting polyubiquitination of K63 junctions and inhibiting the interferon regulatory factor 3 (IRF3) and nuclear factor kappa-B (NF-κB) pathways (17).

#### 2.1.3 ATL3

ATL3 is a power-like GTPase that mainly regulates ER fusion; it is the primary tubular ER-phagy receptor in cells lacking RTN3L, and ATL3 deficiency suppresses tubular ER degradation. ATL3 interacts explicitly with the GABARAP of the ATG8 family through the interaction of two GABARAP motifs, resulting in the sequestration of tubular endoplasmic reticulum in autophagosomes, which are ultimately degraded by lysosomes (18). The process of the unc-51-like kinase 1 (ULK1) complex targeting the ER plays a key role during autophagosome formation, and ATL3 contributes to ULK1 fixation at the specific autophagosome formation site in the ER (19).

#### 2.1.4 TEX264

The N and C termini of TEX264 are hydrophobic and relatively loose fragments, respectively, with a cytosolic gyrase inhibitor (Gyrl)-like structure between the N and C termini. TEX264 is mainly responsible for ER-phagy during starvation. Compared to other ER-phagy receptors, TEX264 is most strongly connected to the LC3, ATG8, and GABARAP autophagy proteins (20). The mechanism by which TEX264 initiates ER-phagy is by using its C-terminal LIR motif to interact with ATG8 in lipid form, and TEX264 then binds to LC3-positive phagosomes. Finally, the autophagosome fuses with lysosomes (21). Excessive ER ribosome volume causes some obstacles to ER-phagy. However, the unique structure of TEX264 solves this problem because its extended C-terminus assists the ER in binding to phagosomes by extending to the cytoplasm and binding to LC3 (22).

#### 2.1.5 CCPG1

CCPG1 is an atypical endoplasmic autophagy receptor that interacts with ATG8 through the typical LIR motif in the cytoplasmic region and binds to FIP200 through the discrete motif (FIR) (23). Zhou et al. also found that the regulation of phosphorylation promotes the interaction of FIP200 with the FIR2 motif of CCPG1 (24). Therefore, it is likely that CCPG1 initiates ER-phagy upon interaction with both proteins. Researchers have found that in addition to the first two autophagy proteins, RB1-inducible coiled-coil (RB1CC1) is also localized to the FIR of CCPG1. Unlike other autophagy receptors, binding to RB1CC1 is required for CCPG1 to trigger ER-phagy (25).

#### 2.1.6 SEC62

SEC62 is a component of the translocon complex and a critical ER-phagy receptor (26). Under ER stress induction, SEC62 binds to the ATG8 autophagy-associated protein to assist in the formation of autophagosomes containing misfolded or unfolded proteins and the ER. Autophagosomes are then transmitted to lysosomes (27). Recent studies have demonstrated that SEC62 not only participates in the UPR, but also improves stress tolerance during the ER stress recovery phase, indicating that SEC62 is a significant autophagy receptor during the ER stress recovery phase (26, 27). The bulk of secretory and membrane proteins is located in the ER and is translocated *via* the SEC61 protein transduction channel. Both SEC62 and SEC63 are associated with SEC61 channels, which form the SEC complex and regulate protein translocation (28, 29).

#### 2.1.7 CALCOCO1

Recently, researchers have found that in addition to the six typical ER receptors in mammals, calcium-binding and CALCOCO1 also mediate ER-phagy as soluble receptors. CALCOCO1 consists of the N-terminal SKIP carboxyl homology (SKICH) domain, the intermediate helix-helix region (CC), and different carboxy-terminal (CT) domains. CALCOCO1 tends to interact with the GABARAP subfamily of ATG8 to mediate macroautophagy. During autophagy, the LIR docking site (LDS) and UIM‐docking site (UDS) are required to stabilize the GABARAP interaction with CALCOCO1, and the UDS sites do not interfere with the role of the LDS sites. CALCOCO1 acts with vesicle-associated membrane protein (vamp)-associated proteins to mediate ER-phagy *via* two phenylalanines (FFs) in an acidic tract (FFAT) motif (30).

### 2.2 Other novel regulatory mechanisms of ER-phagy

**Figure 2:**
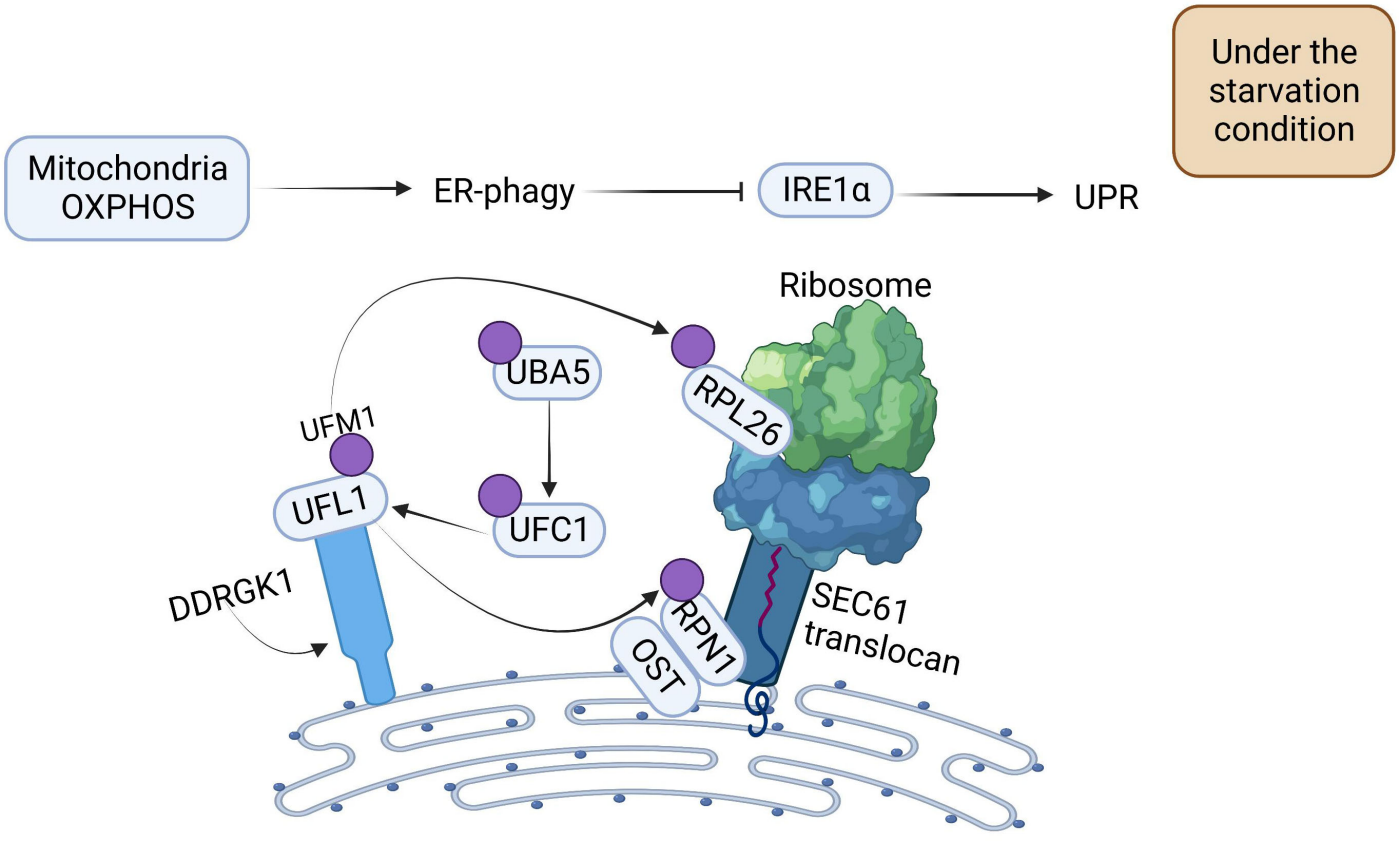
The Mechanism of Oxidative Phosphorylation and DDRGK1-mediated UFMylation Regulation for Propelling ER-phagy. Mitochondria OXPHOS can directly promote ER-phagy. DDRGK1-mediated UFMylation interacts together with E1, E2, and E3. UFL1 delivers UFM1 to the ER surface proteins RPL26 and RPN1, enabling the UFMylation of ER, which then initiates ER-phagy. ER-phagy inhibits IRE1 activity, which in turn blocks the UPR.

With a more comprehensive understanding of the regulatory mechanism of ER-phagy, researchers have found that OXPHOS and DDRGK1-mediated ER surface UFMylation also promote ER-phagy. OXPHOS is positively correlated with ER-phagy. Under starvation conditions, deficiency of OXPHOS severely impedes ER-phagy activation. UFM1-specific ligase 1 (UFL1) is recruited to the ER by DDRGK1, which subsequently impels the activation, conjugation, and ligation of UFM1 to the substrate ribophorin I (RPN1) and ribosomal protein L26 (RPL26) *via* ubiquitin-like modifier activating enzyme 5 (UBA5), ubiquitin-fold modifier conjugating enzyme 1 (UFC1), and the UFL1 cascade, thus triggering ER-phagy to ameliorate the intensity of ER stress (**Figure 2**) (31).

## 3 Potential roles and mechanisms of ER-phagy in ARDS

**Figure 3:**
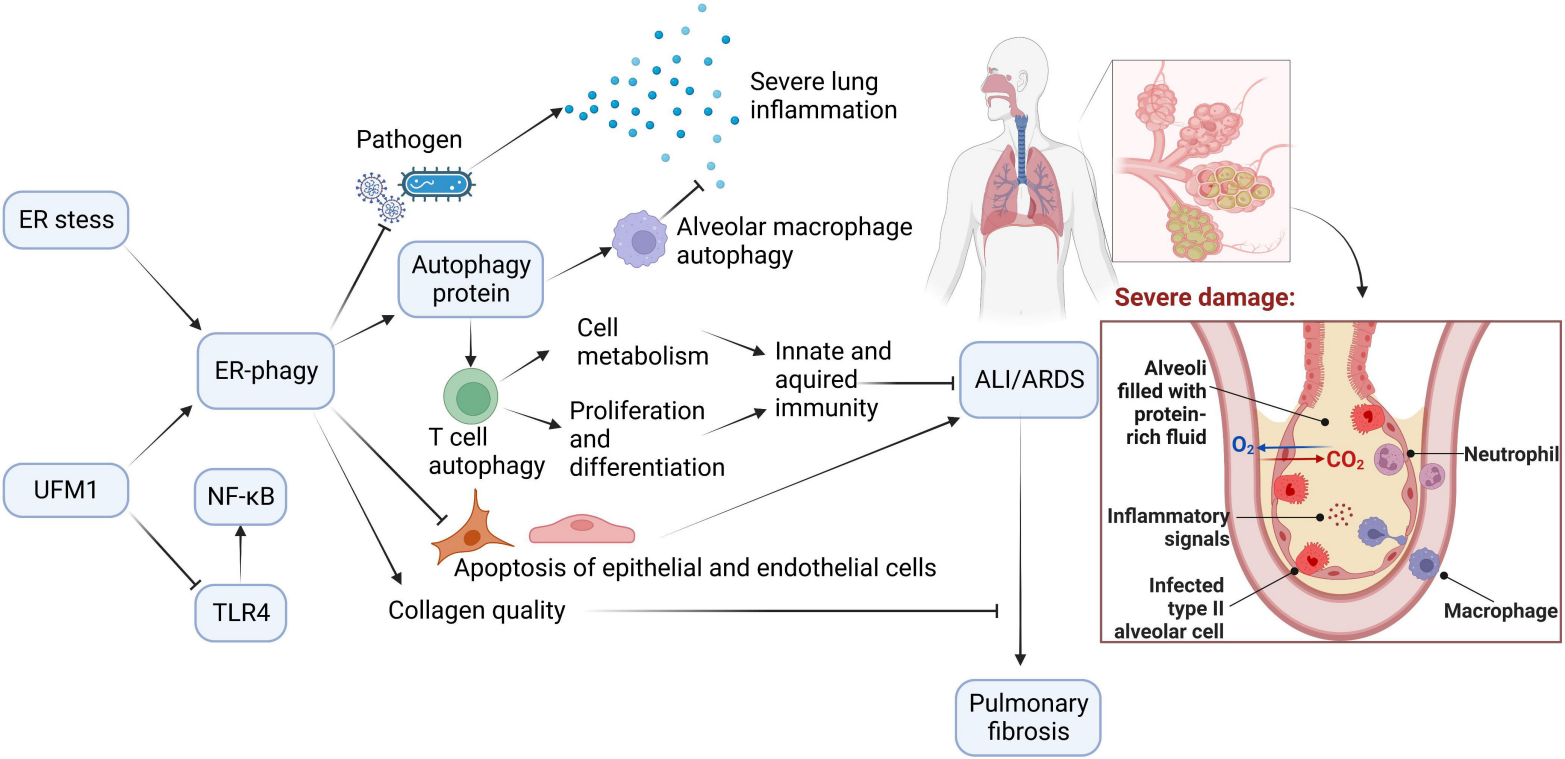
The Potential Role of ER-phagy in ALI/ARDS. Immunity and inflammation are closely associated with ALI/ARDS progression. During the ER-phagy, the autophagy proteins initiate autophagy of immune cells. Autophagy of macrophages reduces inflammation in the lung, and autophagy of T cells promotes cell metabolism, and enhances proliferation and differentiation of cells. ER-phagy can also reduce apoptosis and prevent pathogen infection, which prevent and improve ALI/ARDS. Furthermore, ER-phagy maintains collagen quality and avoids the progression of ALI/ARDS to pulmonary fibrosis.

Autophagy is of great value in delaying ARDS exacerbation. ER-phagy, selective autophagy with high efficiency, plays a crucial role in autophagy. ER-phagy may prevent and ameliorate ARDS induced by different causes by regulating immunity, inflammation, apoptosis, pathogen invasion, and collagen synthesis (**Figure 3**).

### 3.1 ER-phagy regulates immune and inflammatory responses by regulating immune cells, controlling inflammatory material release, and inhibiting variant anti-trypsin and severe pancreatitis

The inflammatory response is the main pathophysiology of ALI/ARDS and the core of ALI/ARDS Pathogenesis (32). Dysregulation of inflammation caused by immune cells is associated with ARDS Pathogenesis (33), and macrophages have been demonstrated to regulate lung inflammation. Macrophages can be polarized into two subsets, namely, M1 and M2. M1 macrophages promote inflammatory responses, while M2 macrophages contribute to the repair of damaged tissue (34). Several studies have demonstrated that the polarization regulation of macrophages plays pivotal roles in various stages of ALI/ARDS (35, 36). Nevertheless, under different circumstances, the properties of diverse macrophage subsets are not rigorously inflexible in each phase of ALI/ARDS (34). Autophagy, responsible for the maintenance of homeostasis, is associated with the polarization of macrophages. Liu et al. found that autophagy promotes the downregulation of inflammation through modulating macrophage polarization in LPS-treated mice (37). Moreover, researchers found that certain drugs use the effect of autophagy on macrophage polarization, thus improving the prognosis of associated diseases (38, 39). For example, Araloside C ameliorates atherosclerosis by regulating macrophage polarization *via* autophagy (38). Whether ER-phagy is involved in the paradoxical regulation of macrophage polarization remains unresolved, whereas researchers suggested that the certain target at ER affects macrophage polarization by activating selective autophagy. For example, due to the enhancement of M2-like macrophage polarization by regulating autophagy *via* NOD-like receptor family, pyrin domain containing 3 (NLRP3) deubiquitination, ubiquitin-specific protease 19 (USP19), an ER-anchored deubiquitinating enzyme, may serve as an important target in treating inflammation-related diseases (40). Autophagy is also closely associated with ALI/ARDS. Lipopolysaccharide (LPS), a common factor inducing ALI/ARDS, may aggravate inflammatory responses *via* inhibition of autophagosome degradation (41). However, several traditional Chinese medicines suppress inflammatory pathway by promoting autophagosome degradation, such as Astragaloside IV, JFNE-A (42, 43). In addition, the certain drug may alleviate ALI/ARDS by modulating macrophage polarization *via* autophagy, such as Sirtuin 6 (SIRT6) (44). Be it autophagy itself or the regulation of macrophage polarization *via* autophagy, all of these mechanisms play critical roles in inflammation. Therefore, the essential contribution of autophagy to immunity is regulating inflammation, and modulating immune cells is involved in the whole process (45).

As a critical type of organelle autophagy, ER-phagy plays a crucial role in alleviating inflammation as well (46). ER-phagy may regulates immune and inflammatory responses by regulating immune cells. Unlike the traditional forms, Anti-PD-L1 (Programmed death ligand 1), a promising measure in treating ALI/ARDS, promotes autophagy by inhibiting the PI3K/Akt/mTOR pathway at the ER in neutrophils, thus attenuating inflammatory responses (47). USP19 anchored at ER alleviates inflammatory responses and damaged tissues by increasing autophagy flux in macrophages (40).Naive T cells differentiate into effector T cells and long-lived memory T cells, which play critical roles in immune responses. As a crucial regulator of T cell quiescence, fully understanding cellular metabolism may be required for developing novel approaches to modulate protective and pathological T cell responses in human diseases (48). T cells require functional autophagy to adapt to activated energy requirements. In T-cell activation and differentiation, autophagy promotes cellular metabolism by degrading cellular components, thus accommodating the energy requirements of activated T cells (49).A previous study has demonstrated that the initiation of ER-phagy by ER stress maintains homeostasis in mature T lymphocytes. Researchers have shown that ER-phagy regulates calcium influx by maintaining ER homeostasis, which subsequently improves T-cell function (50). Recently, the prevalent coronavirus disease 2019 (COVID-19) has been the critical cause of ARDS, and ICU patients with COVID-19 are associated with severe ARDS. Researchers have found that nonsurvivors have significantly increased neutrophils and significantly reduced T cells. Therefore, maintaining homeostasis of T cells is likely to be crucial for preventing and ameliorating ALI/ARDS (51). Although the researches of ER-phagy of immune cells in ALI/ARDS is not very clear, many researchers demonstrated that immune cells maintain homeostasis through autophagy, thus improving the prognosis of ALI/ARDS. Alveolar macrophages are the main immune cells in lung, and researchers have suggested that inducing autophagy in alveolar macrophages significantly alleviates lung inflammation (52). MicroRNAs are associated with the autophagy of alveolar macrophages in LPS-induced ALI/ARDS. Exosomal miR-384-5p target the Beclin-1 autophagy protein to maintain the stabilization of autophagy in alveolar macrophages, thus alleviating lung pathological changes and attenuating inflammatory responses (53). Due to the induction of autophagy of macrophages *via* inhibition of transforming growth factor-β-activated kinase-1-binding protein 2 (TAB2), miR155 becomes a potential candidate for anti-inflammatory therapy during septic lung Injury (54). Moreover, researches demonstrated that some drugs ameliorate ALI/ARDS by activated autophagy of macrophages to mitigate inflammation. SIRT6 induces autophagy of macrophages by activating the AMPK pathway, which leads to M2 macrophage polarization and attenuates inflammation in sepsis-induced ARDS (44). Lipoxins (LXs), synthesized by immune cells such as macrophages and neutrophils, promote anti-inflammatory activities. Researchers showed that LXA4 receptor agonist alleviates ALI-associated inflammation and lung injury by stimulating autophagy (55). Sophoridine may inhibit LPS-induced ALI by enhancing autophagy of macrophages and reducing inflammation (41). At present, the role of autophagy of immune cells in clinical practice is still under research, and these researches mainly focus on immune system diseases. A-synuclein, an autophagy-related marker of peripheral blood lymphocytes, is potentially applied to diagnosis of systemic lupus erythematosus in clinical practice (56). Due to inhibition of over-expansion of activated T lymphocytes *via* autophagy, Tripterygium glycoside fraction n2 (TGA2) provides an effective therapy for inflammatory bowel disease (IBD) (57). These findings also provide important values for exploring functions of ER-phagy of immune cells in clinical practice.

When defending against infection, some immune cells, including macrophages and neutrophils, often cause inflammatory responses in the lung. Pulmonary inflammation is mainly caused by inflammatory factors released from alveolar macrophages. However, the autophagy-related proteins, ATG and FIP200, have been found to maintain innate immune homeostasis, thus avoiding aggravating ALI/ARDS caused by dysregulated inflammation (58). Although ER-phagy effectively regulates lung inflammation, excessive ER-phagy produces side effects. Neutrophils are the first line of defense against pathogen invasion, and the neutrophil extracellular trap (NET) is released to control spread of the pathogen and then kill the pathogen (59). SARS-CoV-2 induces NET release from neutrophils, while excessive NET release causes apoptosis of lung epithelial cells and severe inflammation (60). However, more vigorous activity of autophagy proteins causes more NET release, suggesting that excessive ER-phagy may lead to COVID-19-induced ARDS (60, 61). Oxidative stress and the inflammatory response play vital roles in sepsis-induced ALI/ARDS. Bhattacharya et al. found that the lack of autophagy proteins alleviates oxidative stress and neutrophil-mediated inflammation (62). Therefore, regulation of ER-phagy is needed to alleviate sepsis-induced ALI/ARDS.

Some ER-phagy receptors also initiate ER-phagy to reduce lung inflammation, thus preventing and ameliorating ALI/ARDS. Variant antitryptases cause reduced lung tissue compliance and aggravate lung inflammation. Researchers have found that endoplasmic reticulum to lysosome-related degradation (ERLAD) removes these variant proteins, while the delivery of variant proteins to the lysosome requires LC3 lipidation. FAM134B-mediated ER-phagy degrades the variant of anti-trypsin by binding to lipidated LC3, thus relieving lung tissue inflammation and facilitating gas exchange (63). In the early stage of acute pancreatitis, the local pancreatic tissue releases large amounts of inflammatory cytokines into the bloodstream, which invade the intestine *via* blood flow and damage the intestinal barrier. Increased intestinal wall permeability allows bacteria and endotoxins to enter the bloodstream, causing severe inflammatory responses that disrupt the endothelial barrier (64). Nevertheless, Lahiri et al. found that the exocrine trypsin accumulated in the ER triggers ER stress, and CCPG1-mediated ER-phagy relieves pancreatic inflammation and ER stress, thus avoiding severe pancreatitis-induced ALI/ARDS (65).

In addition, NF-κB plays a fundamental role in inflammatory and immune responses, which is associated with pathogenesis of ARDS. Nimbolide protects against ARDS through suppressing NF-κB nuclear translocation (66). DDRGK1 and UFL1 mediate ER-phagy by interacting with UFM1. Some researches found that DDRGK1 and UFL1, UFM1 are involved in modulating NF-κB signaling. DDRGK1 degrades inhibitors of nuclear factor kappa-B (IκB), resulting in NF-κB nuclear translocation (67). In contrast, UFL1 and UFM1 abrogates NF-κB activation. For example, overexpression of UFM1 suppresses the LPS-induced toll-like receptor 4 (TLR4) pathway, preventing NF-κB nuclear translocation and alleviating the inflammatory response to avoid endothelial damage (68). However, the functional relevance of ER-phagy in NF-κB signaling requires further exploration, which may provide novel approaches to the treatment of ALI/ARDS.

### 3.2 ER-phagy inhibits the apoptosis of alveolar epithelial cells, lung capillary endothelial cells induced by ER stress/mitochondrial dysfunction

In the early stage, UPR maintains cellular homeostasis. However, if unstable ER is persistent, UPR will induce cell death through apoptotic mechanisms, such as PERK- C/EBP homologous protein (CHOP) (69), IRE1- apoptosis signal-regulating kinase (ASK)-Jun N-terminal kinases (JNK) (70). In addition, Calcium also plays a key role in apoptosis triggered by UPR (71). Similar to ER stress, autophagy is a cytoprotective mechanism under nonphysiological conditions. The relationship between autophagy and apoptosis is changeable. Under normal circumstances, autophagy can protect cells from apoptosis. However, prolonged autophagy induced by ER-stress may contribute to apoptosis. In some conditions, apoptosis induced by ER stress even turns to a protective autophagy in resistance to some drugs (72).

ER-phagy is closely linked to ER stress, and the UPR is involved in ER stress triggering ER-phagy (73). For example, Metformin promotes autophagosome formation by activating UPR (74). However, when ER stress loses its balance with ER-phagy, excessive ER stress and ER-phagy induce apoptosis, which exacerbates the progression of ALI/ARDS. Therefore, appropriate regulation of the interaction between ER-phagy and ER stress effectively reduces apoptosis, which may prevent and improve ALI/ARDS. It has been found that both the activation and inhibition of ER-phagy regulate the intensity of ER stress. Liu and colleagues found that Dexmedetomidine induces ER-phagy by activating FAM134B, which subsequently attenuated the ER stress (75). Globular adiponectin has been found to activate SEC62-mediated ER-phagy, thus mitigating ER stress-induced cardiomyocytes apoptosis (76).However, Lim et al. found that palmitate reduces ER stress by inhibiting ER-phagy (77). Persistent ER stress has been demonstrated to cause apoptosis of alveolar epithelial cells, whereas appropriate ER-phagy decreases apoptosis by alleviating ER stress. In acute lung injury models, 4-phenyl butyric acid (4-PBA) triggers ER-phagy to regulate excessive ER stress, attenuating lung inflammation and reducing apoptosis of alveolar epithelial cells (78). As a scavenger of reactive oxygen species (ROS) and inducer of antioxidant systems, Melatonin suppresses ER stress by restoring autophagic flux, thus attenuating apoptosis and lower infiltration of inflammatory cells in lung (79). Therefore, Melatonin may contribute to ameliorate ALI/ARDS by regulating interaction between ER stress and ER-phagy. In addition to persistent ER stress leading to apoptosis, inhibition of ER-phagy also activates the apoptosis pathway in certain conditions (80).

Mitochondrial dysfunction exacerbates hyperoxia-induced damage to alveolar epithelial cells (81). As the most important organelles in the cell, the ER and mitochondria have tight junction structures known as mitochondria-associated ER membranes (MAMs). Recently, the endoplasmic reticulum-mitochondria encounter structure (ERMES) has been demonstrated to connect the ER and the mitochondria, facilitating the exchange of material between the two organelles. However, misfolded proteins accumulated in the ERMES cause mitochondrial dysfunction (82). Kellner et al. found that heavily accumulated ROS induces oxidative stress, which later causes ALI/ARDS by increasing the apoptosis of lung capillary endothelial cells (83). Chen et al. found that impaired mitochondria produce excessive ROS. Nevertheless, ER-phagy has been demonstrated to inhibit apoptosis due to oxidative stress and ER stress in other tissues. In the model of mitochondrial injury, Panax notoginseng saponin (PNS) attenuates ROS-mediated ryanodine receptor 2 (RYR2) oxidation, subsequently inhibiting ER stress-induced apoptosis *via* ER-phagy (84). Luo et al. found that the accumulation of AGEs activates the ROS pathway, which initiates FAM134B-mediated ER-phagy and then impedes the apoptosis of nucleus pulposus cells (85).

Although the apoptotic effect of ER-phagy on ALI/ARDS has not yet been determined, researchers have demonstrated that ER-phagy is closely associated with lung diseases, which plays crucial roles in cellular survival. As inducers of ER-phagy, both FAM134B-1 and FAM134B-2 isoforms are positive in normal lung tissue, whereas a significant downregulation of FAM134B is observed in lung cancer (86). In addition, many researches proved that autophagy is involved in the progression of ALI/ARDS by regulating apoptosis. Epithelial cell dysfunction, such as Alveolar type 2 (AT2) cells, has emerged as a critical part of the pathophysiology of diffuse parenchymal diseases. Quality control systems, including UPR and autophagy, are essential to avoid the occurrence of ALI/ARDS. AT2 cells maintain homeostasis by selective removal of impaired cellular organelles *via* autophagy, such as ER or lysosome-related organelles (87). The oxidative stress is associated with ROS and autophagy, and autophagy is seen as a secondary defense of oxidative stress. Beyer et al. found that autophagy ameliorates dysfunctional mitochondria, thus restoring barrier integrity by regulating apoptosis of lung microvascular endothelial cells in ROS-induced lung injury (88). Certain drugs, such as Genipin, inhibit apoptosis by mitigating oxidative stress and mitochondrial damage via activation of autophagy in LPS-induced ALI (89).

Besides, apoptotic alveolar macrophages releases more ROS and exacerbates inflammatory cascading, resulting in apoptosis of alveolar epithelial cells and aggravated lung injury (52, 90). However, autophagy of alveolar macrophages contributes to maintain cellular homeostasis and ameliorate lung injury by regulating apoptosis. Fan et al. found that autophagy of alveolar macrophages reduces alveolar macrophage apoptosis by mitigating endoplasmic reticulum stress and oxidative stress (91). MiR−223−3p−loaded exosomes activates alveolar macrophage autophagy and restores anti-apoptotic effects by targeting serine/threonine kinase 39 (STK39) in the lung tissue of ALI mice (92). Meanwhile, the integrity of degradation is essential for the autophagy process. Under reduced lysosome conditions, LPS promotes the accumulation of autophagosomes in alveolar macrophages, leading to severe inflammation and apoptosis of alveolar macrophages (90). Certain drugs can alleviate ALI by suppressing apoptosis of alveolar macrophages *via* autophagy of alveolar macrophages, and some of them contributes to alleviate lung inflammation as well, including LXA4 receptor agonist (55). Nevertheless, Complement C5a exacerbates alveolar macrophage apoptosis *via* enhancement of autophagy in acute lung injury (93).

ER-phagy is triggered by mediating ER surface UFMylation *via* activated UFM1. IRE1α cleaves the X-box-binding protein 1 (XBP-1)-encoded mRNA *via* endonuclease to produce the active transcription factor XBP1, thus initiating the UPR. XBP-1 induces the upregulation of UFM1 expression by inhibiting vesicular trafficking (94). The translocation of UFM1 to the ER has been found to be dependent on UFM1-binding protein 1 containing a PCI domain (UFBP1). Under ER stress, UFM1 and UFBP1 are upregulated and reduce apoptosis, suggesting that UFM1-mediated ER-phagy may protect tissue injury by inhibition of apoptosis (95).

### 3.3 ER-phagy avoids the proliferation and transmission of pathogens

Pathogen infection is a common cause of ALI/ARDS, and ER-phagy is closely related to pathogenicmicrobial clearance. ER-phagy may prevent infection by activated ER-phagy receptors or regulating immune cells, thus preventing or ameliorating the development of ALI/ARDS.

ER-phagy receptors, including FAM134B, ATL3, SEC62, and others, control viral proliferation and transmission. Viral invasion of lung tissue leads to damaged vascular endothelium, the body then initiates FAM134B-mediated ER-phagy and reduces the supply of raw materials and vesicle membranes required for viral replication, thus inhibiting viral proliferation and transmission (13). ATL3 interacts with the viral capsid, preventing virus assembly and transport, and ATL3 cleaves the spike protein on the immature SARS-CoV-2 surface by activating the Flynn protease (96). Upon activation of SEC62 *via* viral infection, activated SEC62 drives IRE1α phosphorylation, which subsequently induces the IRE1-JNK pathway and delivers autophagosomes to lysosomes, thus mitigating virus-induced ER stress and impeding viral replication (97). However, RTN3 endures viral proliferation, facilitating the virus to escape from the ER to the cytoplasm by reshaping the ER membrane to make it more flexible (98). Usually, viruses are cleared by ER-phagy, but coronaviruses inhibit autophagosome formation through related proteases or their specialized structures, resulting in impaired ER-phagy (99). ER-phagy, mediated by ER-phagy receptors, is also associated with fungal infection. For example, in response to A.fumigatus stimulation, the levels of CCPG1 expression are increased to a large extent. CCPG1-mediated ER-phagy removes pathogens and suppresses excessive inflammatory responses, thus avoiding aggravating ALI/ARDS caused by pulmonary fungal infection in the immunocompromised setting (100).

Many researches have shown that autophagy of immune cells plays a vital role in improving the prognosis of ALI/ARDS, such as alveolar macrophages. Due to the effective removal of intracellular pathogens and subcellular organelles (including ER) through activation of autophagy, STING was initially viewed as a crucial molecule in immunity. However, a recent research has demonstrated that in alveolar macrophages, STING inhibits autophagic flux by perturbing lysosomal digestion, thus leading to sepsis-induce ALI (101). Autophagy dysfunction induced by prostaglandin E2 (PGE2) contributes to survival of P. aeruginosa in alveolar macrophages, thus increasing the occurrence of ALI in P. aeruginosa-infected bone marrow transplant (BMT) mice (102). Defective autophagy leads to depletion of ATP synthesis by severe mitochondrial dysfunction, then significantly decreases antimicrobial activity. However, researchers have found that AMPK- peroxisome proliferator-activated receptor-gamma, coactivator 1α (PPARGC1A) axis promotes antimicrobial host defense through activation of autophagy in alveolar macrophages (103). Although the direct effect of ER-phagy of immune cells on pathogen infection is not largely explored, ER-phagy may regulate the proliferation and differentiation of immune cells through autophagy proteins. Catechins activate CD8+ T cytotoxic lymphocytes by upregulating the protein levels of Beclin-1 and Atg5-Atg12, thus enhancing the adaptive immunity against viral infections (104). ATG proteins inhibit activated CD8+ T cells from converting into effector cells, whereas autophagy also promotes the transformation of effector cells into memory cells during this process, which improves the quality of memory cells and strengthens the host defense mechanisms (105). Pei and colleagues found that ATG proteins are associated with the proliferation and differentiation of NKT cells. The deletion of ATG proteins causes a corresponding decrease in NKT cells, which weakens both innate and acquired immune function. Due to the limitation of resisting pathogen infection effectively, infection-induced ALI/ARDS is more easily caused (106).

In addition to clearance of virus and fungus, ER-phagy is also responsible for eliminating bacteria. Bacteria survive and proliferate in host cells, which depend on a safe and nutritive environment. The ER has such conditions, which encourage bacteria to stabilize and multiply inside the cell (107). These bacteria are prone to trigger ER stress. As a result, ER-phagy is initiated, immediately eliminating bacteria and maintaining ER homeostasis (108). Researchers have demonstrated that when infected by gram-positive bacteria, the defensive machinery activates ER-phagy by inhibiting mTOR activity. ER-phagy reduces PERK and CHOP expression, and it improves the immune system to enhance the defensive function of the host (109).

### 3.4 ER-phagy prevents the progression of ALI/ARDS to pulmonary fibrosis by controlling collagen synthesis

If the pathological changes of ALI/ARDS are not corrected immediately, severe pulmonary fibrosis, the major cause of mortality in ARDS patients, will occur in the later stage to aggravate the development of the disease (110). Some researchers have attempted to restore the integrity of alveolar-capillary barrier by two molecules, platelet-endothelial cell adhesion molecule-1 (PECAM1) and wingless-related integration site (Wnt), thus attenuating diffuse alveolar damage and preventing fibroproliferative ARDS (111).

In addition to inducing apoptosis in lung epithelial cells, persistent ER stress also exacerbates lung fibrosis. ALI appears in bleomycin-treated mice, and palmitate increases apoptosis of lung epithelial cells, which exacerbates endoplasmic reticulum stress by upregulating the expression of UPR proapoptotic proteins, resulting in the progression of ALI/ARDS to pulmonary fibrosis (112). Moreover, researches have shown that ER stress promotes the development and progression of pulmonary fibrosis by the regulation of alveolar epithelial cell (AEC) apoptosis, epithelial–mesenchymal transition, and myofibroblast differentiation (113). Therefore, controlling the intensity of ER stress may be beneficial for the prognosis of ALI/ARDS through reduced pulmonary fibrosis.

ER stress is closely associated with autophagy, and some drugs-mediated protection against pulmonary fibrosis through regulation of ER stress and autophagy. For example, Spermidine has been shown to resolve fibrosis by inhibiting ER stress and enhancing autophagy. Thus, autophagy also plays a critical role in suppressing fibrotic lung. Recently, researches demonstrated that defective autophagy contributes to excess production of extracellular matrix and alteration of fibroblasts to myofibroblasts (114). Yang et al. found that Oridonin attenuates LPS-induced early pulmonary fibrosis by restoring impaired autophagy (115). Therefore, high-quality autophagy may prevent ALI/ARDS from progressing to pulmonary fibrosis. As a cellular organelle with efficient autophagy, ER has been demonstrated to be involved in a novel anti-fibrotic function (116). Excessive deposition of extracellular matrix, mainly collagen protein, is the main factor leading to pulmonary fibrosis. However, ER-phagy maintains procollagen quality to prevent fibrosis due to intense ER stress. Because collagen synthesis is complex and prone to errors, FAM134B-mediated ER-phagy degrades misfolded collagen, avoiding intense ER stress due to the accumulation of these proteins. Researchers found that the ER-resident lectin chaperone protein, CANX, and the ER phagocytic receptor, FAM134B, are pivotal in this process. CANX, a coreceptor for FAM134B-mediated ER-phagy, is responsible for binding and recognizing misfolded procollagen in the ER lumen. FAM134B then binds to the LC3 autophagic protein and transports the ER containing CANX and procollagen to lysosomes for degradation (117). FAM134A and FAM134C also function in maintaining collagen homeostasis. Therefore, deficiency of the FAM134 protein inhibits ER-phagy, thus causing a significant increase in collagen and leading to a dramatically increased incidence of pulmonary fibrosis (118).

Apart from the regulation of immunity, inflammation, apoptosis, and pathogen infection, autophagy of immune cells also plays an important role in pulmonary fibrosis. Aggregated macrophages in damaged lung areas may aggravate inflammation and accelerate fibrogenesis, whereas Dioscin decreases chemokines and proinflammatory cytokines secretion by inducing autophagy of alveolar macrophages, thus avoiding pulmonary fibrosis due to collagen deposition (52). Trehalose exerts antifibrotic effects by restoring lysosomal function, accelerating autophagic substrate degradation, and enhancing autophagic flux in Crystalline Silica (CS)-treated Alveolar Macrophages (119). Nevertheless, Liu et al. found that TP53-upregulated modulator of apoptosis (BBC3) triggers the release of pro-fibrotic cytokines by enhancing autophagy of alveolar macrophages (120).

## 4 Conclusions and perspectives

ALI/ARDS, an intractable inflammatory disease in ICU, has a high mortality rate. It is urgent to find the treatment for ALI/ARDS, and the key is to understand the pathogenesis. However, the pathogenesis of ARDS is not elucidated up to now.

ER-phagy was discovered in 2007, and researchers began intensively studying ER-phagy through ER-phagy receptors in 2015. Therefore, as an important organelle autophagy, ER-phagy is a relatively new field of research. Recently, researchers have conducted many studies on ER-phagy. When ER stress asts too long, and the proteasome degradation system is insufficient to compensate, ER-phagy is mediated by different ER-phagy receptors or other pathways, resulting in removal of damaged ER fragments, effectively improving the intracellular environment. In other damaged tissues, since ER-phagy is involved in the regulation of inflammation and apoptosis, it is regarded as an important therapeutic target of associated diseases.

ALI/ARDS is characterized by complex pathological changes, and inflammation and apoptosis play crucial roles in each pathological stage. Although the specific mechanisms of ER-phagy in ALI/ARDS have not yet be determined, ER stress and autophagy have been proved to be involved in the progression of ALI/ARDS. Researches demonstrated that ER stress is a vital pathogenesis leading to ALI/ARDS, and this damage can be reversed by autophagy *via* activation or inhibition of related pathways. ER-phagy may be involved in the different etiology-related ARDS by regulating immunity and inflammation, modulating apoptosis, preventing microbial infection, and preventing lung fibrosis. Besides, immunity and inflammation are the most important pathogenesis of ALI/ARDS, and immune cells are essential to the immune and inflammatory responses. Although ER-phagy of immune cells is not largely explored, the autophagy of immune cells does play critical roles in ALI/ARDS through affecting four aspects, including immunity and inflammation, apoptosis, microbial infection, and fibrosis. Many researches have shown that autophagy of immune cells can ameliorate ALI/ARDS. However, several studies suggested that under certain circumstances, autophagy has a detrimental role in ALI/ARDS pathogenesis, which is related to the primary etiology, the cell types, the stage of ALI/ARDS. Therefore, further study on ER-phagy of immune cells is a promising measure to resolve problems of therapies. As for the detail directions of researches, researchers can mainly focus on the relevance of ER-phagy in macrophage polarization and inflammatory signals in the future.

Existing studies have demonstrated that ER-phagy is pivotal for improving the prognosis of many diseases. Accordingly, conducting experiments on the effects of ER-phagy in ALI/ARDS may provide significant values in developing novel therapies for ALI/ARDS.

## Author contributions

SL, XF, RZ, JZ, HW, JL, CW, LW, LZ wrote and reviewed the manuscript. SL prepared the figures. SL and XF discussed the ideas in the draft. LZ reviewed the manuscript and provided suggestions. All authors contributed to the article and approved the submitted version.
